# Enhanced water absorption of tissue paper by cross-linking cellulose with poly(vinyl alcohol)

**DOI:** 10.1007/s11696-022-02188-y

**Published:** 2022-04-08

**Authors:** A. Cláudia S. Ferreira, Roberto Aguado, Raquel Bértolo, Ana M. M. S. Carta, Dina Murtinho, Artur J. M. Valente

**Affiliations:** 1 CQC, Department of Chemistry, University of Coimbra, 3004-535 Coimbra, Portugal; 2 LEPAMAP-PRODIS Research Group, University of Girona, C/ M. Aurèlia Capmany, 61, 17003 Girona, Spain; 3 RAIZ - Forest and Paper Research Institute, Quinta de S. Francisco, Apartado 15, 3801-501 Aveiro, Portugal

**Keywords:** Cellulose, Cross-linking, Kraft pulp, Pulp and paper, Poly(vinyl alcohol)

## Abstract

**Abstract:**

Tissue paper was the only paper grade whose consumption increased during 2020 in Europe. In a highly competitive context, this work explores a strategy based on bisacrylamide cross-linkers and poly(vinyl alcohol) (PVA), seeking to enhance the water uptake of pulps for tissue paper and the key properties of the resulting tissue sheets: water absorption capacity, capillarity, softness, porosity, and strength. For that, α-cellulose from cotton and a kraft hardwood pulp, in parallel, were reacted with *N,N’*-methylenebisacrylamide, both in the absence and in the presence of PVA. The water desorption rate of the modified polymers was monitored. Pulp blends were then mixed with a conventional softwood pulp (30%) to prepare laboratory tissue paper sheets (20 g m^–2^). For cotton cellulose, cross-linking with PVA more than doubled the water uptake, up to 7.3 g/g. A significant enhancement was also obtained in the case of pulps, up to 9.6 g/g, and in the case of paper, to 11.9 g/g. This improvement was consistent with a drastic increase in porosity, and it was not detrimental to paper strength.

**Graphical Abstract:**

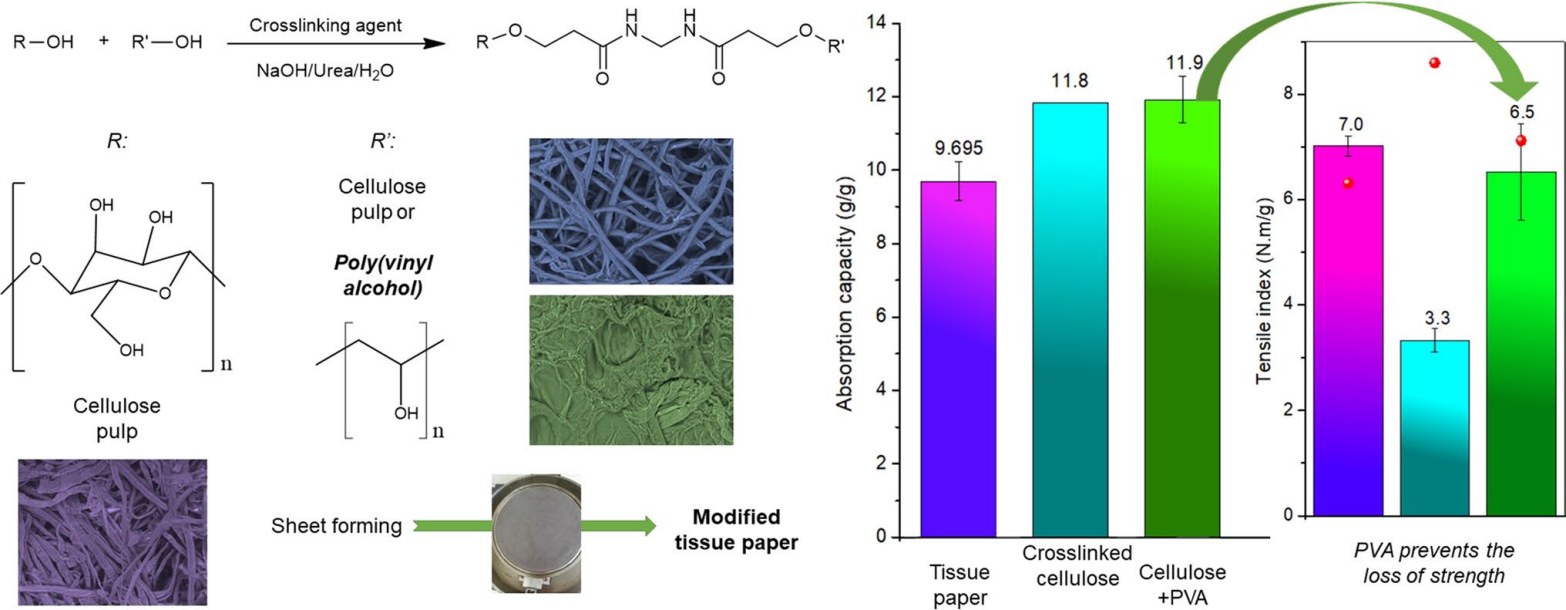

## Introduction

Tissue paper is expected to reach a business volume of USD 102.57 billion by 2026, accounting for an average annual growth of USD 5.27 billion (Fortune Business Insights 2020). In 2020, while the European consumption of graphic paper decreased by 18%, partly because of the COVID-19 crisis and by replacement with digital communication, paper for sanitary and household consumption showed an increase of about 4% (CEPI 2021). Besides the conventional usage, for instance, bath and toilet paper, facial tissue, and paper towel, alternative applications include the development of sensors (Zhao et al. 2021) and catalysts (Li et al. 2015), and its role as a separation membrane (Roy et al. 2018) or as an adsorbent for wastewater treatment (Khan et al. 2020). For all the aforementioned applications, a balance between several properties (high water uptake, softness, low basis weight, and enough strength) is required (Vieira et al. 2020; Morais et al. 2020).

In both household and commercial contexts, enhancing water absorption without loss of mechanical properties is probably the biggest challenge that tissue papers manufacturers still face (Olejnik et al. 2019). This is usually achieved through various approaches, including modifications in the pulp composition, the use of additives (Guan et al. 2019), and different processing methods (Naithani et al. 2020; de Oliveira Mendes et al. 2020). In this paper, we report a new approach based on the chemical modification of cellulose with poly(vinyl alcohol) (PVA). This polyol is a water-soluble, non-toxic, non-carcinogenic, biodegradable, cheap, and easily processed polymer. PVA can form physically and chemically cross-linked hydrogels that exhibit a high degree of swelling in water and a rubbery and elastic nature (Hassan and Peppas 2000; Date et al. 2020). It is also used as an additive in many formulations due to its amphiphilic and neutral properties (Baptista et al. 2016b; Knaapila et al. 2016). Additionally, due to its high density of hydroxyl groups, PVA is easily grafted onto other polymers (Filho et al. 2018).

There are several ways to covalently cross-link hydroxyl groups. These include polycarboxylic acids toward ester bonds (Aguado et al. 2021), but aqueous media are generally excluded in this case. The use of glutaraldehyde is ubiquitous for peptide cross-linking, but, in the case of hydroxyl groups, the formation of hemiacetal bonds is reversible and requires acid catalysts (Priya et al. 2014). *N,N’*-methylenebisacrylamide (MBA), also widely used (Alpaslan et al. 2021), stands as a wise choice of cross-linker due to its reactivity and its compatibility with water.

All considered, it is hypothesized that the concurrence of PVA–cross-linker–cellulose bonds, PVA–cross-linker–PVA bonds, and cellulose–cross-linker–cellulose bonds prompt the generation of a highly water-sorbent network, boosting intra-fiber and inter-fiber sorption at the same time. In order to test this hypothesis, this work involved the synthesis and characterization of PVA–cross-linked α-cellulose and hardwood pulp, additionally performing the same cross-linking reaction without PVA (i.e., only between cellulose chains). Reagent proportions were carefully selected so that water sorption was boosted without compromising proper drainage during sheet formation. The effect of PVA molecular weight on the water uptake (equilibrium and kinetics), among other physical and chemical properties, was also assessed. The cross-linked paper pulp with the best water uptake properties was used for the preparation of tissue paper samples. Papermaking properties of lightweight sheets were then evaluated in terms of water absorption capacity, capillarity, tensile index, softness, and permeability to air.

## Experimental

### Materials

α-Cellulose from cotton (powder, > 50% of fibers being below 150 µm long) was purchased from Sigma-Aldrich, while commercial bleached eucalyptus kraft pulp (BEKP) and commercial bleached softwood kraft pulp (BSKP) were provided by a Portuguese tissue mill. PVA with two different molecular weights (M_w_ ca. 61,000, 98.0 mol% hydrolysis and M_w_ ca. 27,000, 98.0 mol% hydrolysis) was obtained from Sigma-Aldrich.

Urea (99.0%), sodium hydroxide, and MBA (99%) were purchased from BDH Chemicals, José Manuel Gomes dos Santos, and Merck, respectively. A dialysis tubing cellulose membrane (molecular weight cutoff, 14,000 Da) was acquired from Sigma-Aldrich. Millipore-Q water was used in all experiments.

### Cross-linking reactions

The methodology used for the incorporation of PVA into α-cellulose and BEKP, by cross-linking, was based on a previously reported procedure (Geng 2018), although with certain modifications; the method details, considering the use of α-cellulose, follow. Initially, α-cellulose was dispersed in pre-cooled mixtures of 7/13/100 NaOH/urea/water (w/w/v) and stirred, in an ice bath, until homogenization. In parallel, an aqueous solution of PVA (2%, w/w) was prepared by dissolving the polymer at 90 °C, under continuous stirring and reflux conditions. Once cooled down, NaOH and urea were added to obtain a final PVA/NaOH/urea/water ratio of 2/7/13/100 (w/w/w/v). Then, both polymer solutions were mixed (50/50, v/v), so that the total polymer concentration is 2% (w/w) in all cases. Then, the cross-linker was added (0.2 g and 0.5 g per gram of polymer), and the reaction mixture was stirred for 2 h. While higher MBA dosages would increase swelling to larger extents, as already shown for cellulose without PVA (Geng 2018), this work does not seek a superabsorbent hydrogel, but a paper-compatible additive that does not hinder drainage during sheet formation.

**Scheme 1:**
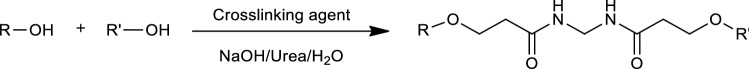
Generic reaction of MBA-mediated crosslinking between two hydroxy polymers

Cross-linking took place according to the reaction shown in Scheme 1. The mixture was then placed in an oven at 60 °C for 12 h. Using the dialysis membrane (cutoff 14,000 Da), cross-linked samples of PVA and cellulose were soaked in an excessive amount of deionized water, which was repeatedly replaced with freshwater until the pH reached 7.

The same method was performed for BEKP. Likewise, for comparison purposes, cross-linking between cellulose chains was also carried out. In this case, MBA was directly added onto the alkaline solution of α-cellulose or BEKP, without the addition of PVA.

### Characterization of the modified celluloses

The incorporation of PVA into α-cellulose and BEKP was qualitatively confirmed by ATR-FTIR spectroscopy using a Varian Cary 630 spectrometer. Spectra were recorded using oven-dried samples as they were obtained.

Thermogravimetric analysis (TGA) was carried out by means of a TG209 F3 Tarsus thermogravimetric analyzer (NETZSCH Instruments). Samples (ca. 5 mg) were heated at 10 ºC min^−1^ from 25 to 600 ºC, under nitrogen, at a flow rate of 20 mL min^−1^.

Backscattered electron imaging was performed using a TM4000Plus scanning electron microscope (SEM) with EDS (Hitachi), operating at an acceleration voltage of 15 kV.

Cross-linking of α-cellulose and BEKP with PVA was also assessed by the ability of the materials to sorb and desorb water. Thus, the water uptake was measured by following a methodology adapted from the DIN standard 53,814. Briefly, the method consists of immersing a sample in water which is then left to swell overnight. Afterward, the samples were centrifuged at 3000 rpm, for 30 min, in an IEC Centra-3C centrifuge. The excess water was removed, the samples were weighed, and the mass was recorded, *m*_eq_. The samples were then dried in an oven, at 60 °C, until constant weight, *m*_0_. In each of the experiments, which were carried out in quadruplicate, the water uptake (%) was calculated using Eq. 1:

1 $$Water\;uptake\;\left( \% \right) = \left( {m_{eq} - m_{0} } \right) \times 100/m_{0}$$

### Papermaking properties

Using the modified BEKP, laboratory handsheets with low basis weight (20 g/m^2^) were prepared (Table 1), according to an adaptation of ISO 5269–1 standard, and using 70% of modified BEKP with 30% of BSKP. For the sample coded as B2, and in light of the results from the characterization of blends, the PVA used was that of *M*_*w*_ 27,000 g mol^–1^ (PVA 27 k). The handsheets obtained for each formulation were air-dried under controlled temperature (23 ± 1 °C) and relative humidity (50 ± 2%) conditions. Subsequently, their tissue properties were monitored. Basis weight and bulk were determined following ISO 12625:6 and 12,625:3, respectively, while the tensile index was determined in a vertical tensile tester (Frank-PTI), according to ISO 12625:4. Softness was analyzed on a tissue softness analyzer (TSA) from Emtec, a device comprising an internal method that estimates softness as handfeel (HF). The capillary rise was evaluated according to an adaptation of ISO 8787 on an Enrico Toniolo apparatus. The water absorption capacity was performed in quadruplicate on a Frank-PTI basket immersion equipment, following an adaptation of ISO 12625–8 standard. Finally, air permeability was tested using a FX3300 LabAir III equipment (FEXTEST Instruments).

**Table 1:** Handsheets formulations in terms of mass of BEKP, BSKP, cross-linked BEKP, and cross-linked blends of BEKP + PVA (modified fibers)

Formulations	B0	B1	B2
BSKP (%)	30	30	30
BEKP (%)	70	–	–
Modified fiber (%)	0	70	70

B1: cross-linked BEKP; B2: cross-linked blends of BEKP + PVA 27 k

## Results and discussion

### α-Cellulose-PVA cross-linked blends

**Fig. 1:**
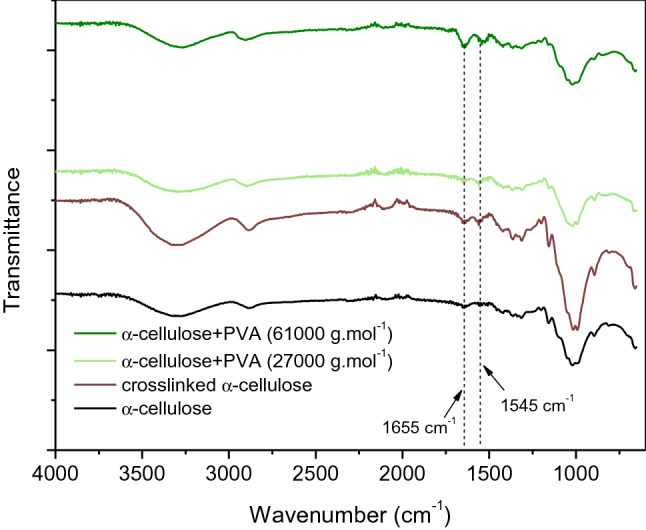
FTIR spectra of α-cellulose and cross-linked samples of α-cellulose and α-cellulose–PVA, using 0.5 g of MBA per gram of polymer

The study of the effect of molecular weight of PVA on the properties of the handsheets formulations was preceded by the characterization of the modified celluloses, obtained by cross-linking of α-cellulose with different PVA samples using *N,N’*-methylene-bis-acrylamide (MBA) as cross-linking agent. PVA with different molecular weights (*M*_*w*_ ca. 61,000, 98.0 mol% hydrolysis; and *M*_*w*_ ca. 27,000, 98.0 mol% hydrolysis) was used. For comparative purposes, a cross-linked sample of α-cellulose with MBA was also prepared. The obtained cross-linked blends were characterized by FTIR (Fig. 1). From the analysis of Fig. 1, it can be observed, in all cross-linked cellulose samples, the presence of two vibrational modes at 1655 cm^−1^ and 1545 cm^−1^, which can be assigned to the presence of carbonyl groups (from PVA acetate residual groups and MBA) and –NH groups (from MBA), respectively.

**Fig. 2:**
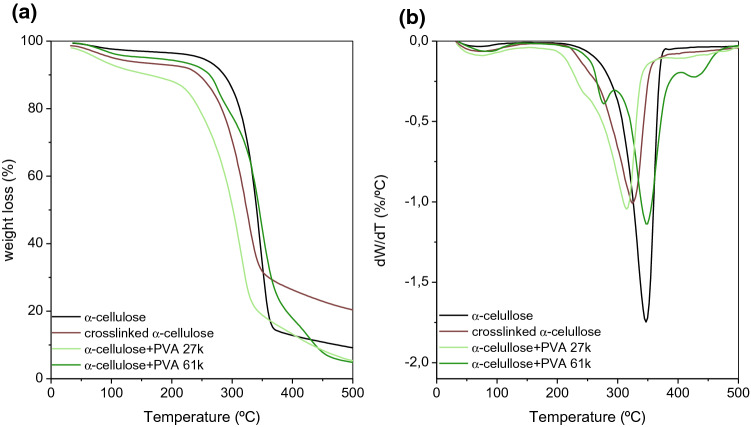
Thermograms (**a**) and DTG curves (**b**) for the neat and cross-linked α-cellulose polymers

The thermograms and corresponding dTGs of neat α-cellulose and cross-linked celluloses are shown in Fig. 2. It can be seen that below 100 °C some weight loss occurs, due to the release of moisture. At *T* > 100 °C, temperatures of maximum degradation rate (*T*_m_) were computed as the minimum in the derivative curve, and the results are summarized in Table 2. For α -cellulose, only one degradation step was observed with a maximum temperature at *T*_m,2_ = 347 °C, in agreement with values reported in the literature (I.P. et al. 2019; Shaikh et al. 2021). However, upon cross-linking, the *T*_m,2_ of the α-cellulose decreases about 20 °C. This can be justified either by the increase in the mechanical stress of the polymer induced by cross-linking and/or by a decrease in the fraction of the crystalline polymorph cellulose I toward cellulose II and amorphous cellulose (Udoetok et al. 2018). Two additional steps, with maximum degradation temperatures labeled as *T*_m,1_ and *T*_m,3_, were observed for PVA-containing blends: The first one was only clearly observed for the higher molecular weight PVA at *T*_m,1_ = 277 °C being assigned to the loss of water bound (non-freezing water) bound to the polymer network (Yang et al. 2009), and the latter occurred at around 428 °C and can be justified by the loss of PVA crystallinity (Baptista et al. 2016a). It was observed that for lower molecular weight PVA blends, *T*_m,2_ was significantly lower than the degradation temperature for α-cellulose and lower than for cross-linked α-cellulose. Such a decrease can be justified by either a plasticization effect or just an overlap between the loss of bound water, characteristic of PVA thermograms, and cellulose (Filho et al. 2018; Udoetok et al. 2018). It is also worth noticing that the *T*_m,3_ increased by increasing the PVA molecular weight; i.e., the order of PVA structure increases by increasing the molecular chain length (Hdidar et al. 2017).

**Table 2:** Maximum degradation temperatures (*T*_m_) obtained by thermogravimetric analysis, for α-cellulose and α-cellulose–PVA blends, cross-linked with 50% MBA (on the basis of dry polymer weight)

	T_m,1_/°C	T_m,2_/°C	T_m,3_/°C
α-cellulose		347	
Cross-linked α-cellulose		325	
α-cellulose + PVA 27 k		315	400
α-cellulose + PVA 61 k	277	348	428

**Fig. 3:**
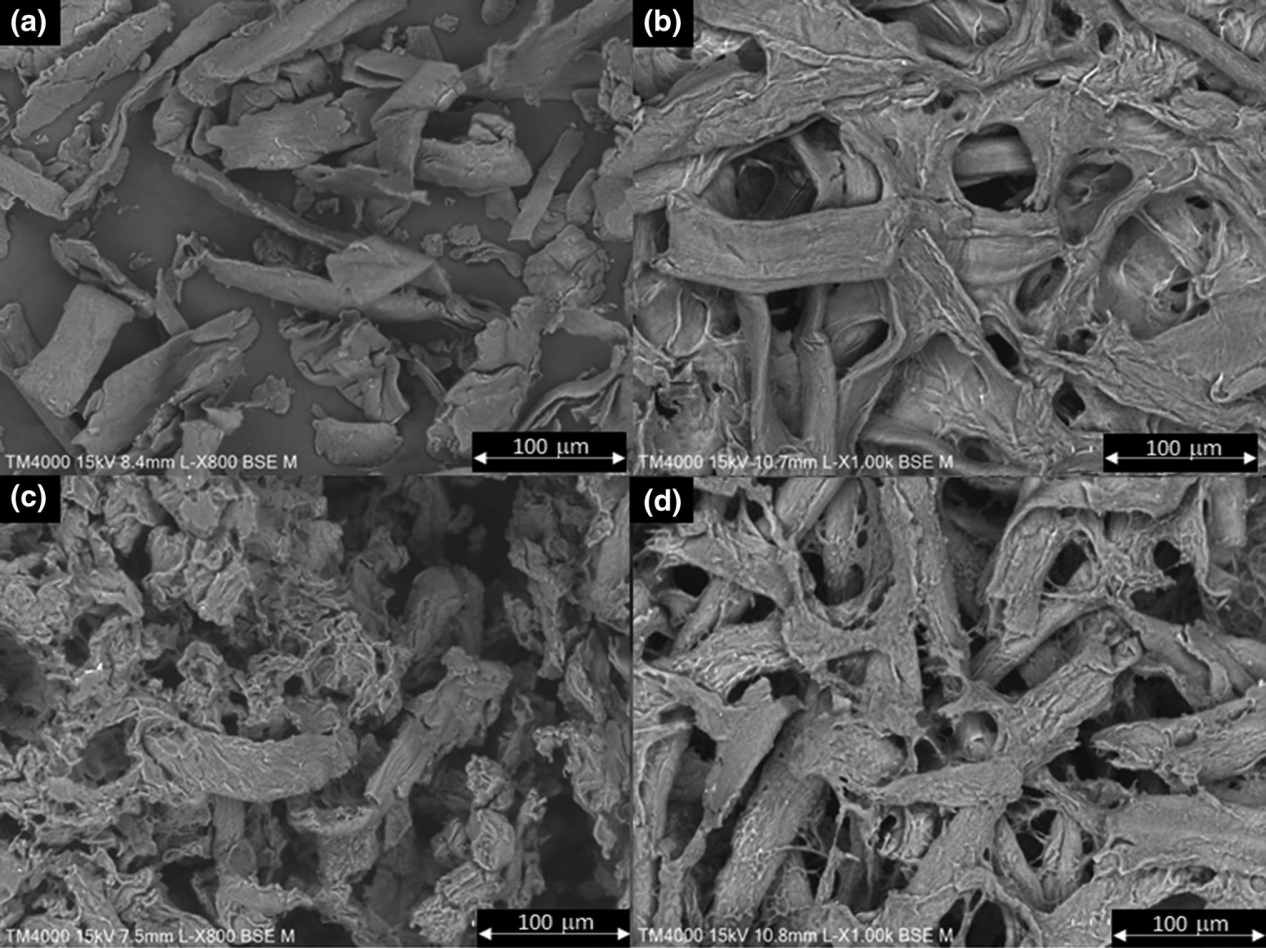
SEM images of α-cellulose (**a**), cross-linked α-cellulose (**b**), α-cellulose + PVA 27 k (**c**) and α-cellulose + PVA 61 k (**d**). Magnification × 1000

**Fig. 4:**
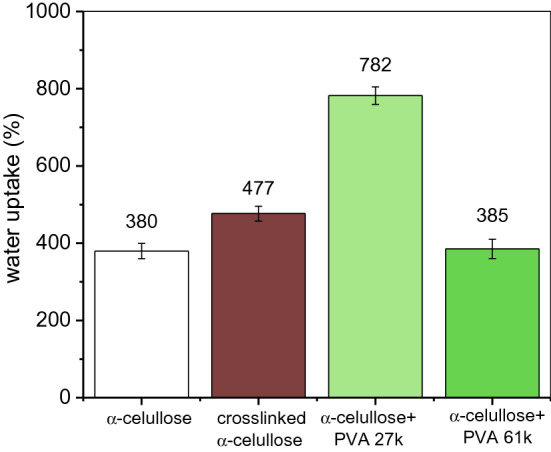
Water uptake for the different α-cellulose-based polymers. The amplitude of the tolerance intervals equals twice the standard deviation

Significant modification of the α-cellulose and α-cellulose + PVA morphology upon cross-linking can also be observed by SEM (Fig. 3). Cross-linking of α-cellulose led to the formation of an entangled, wide ribbonlike structure with high surface porosity (Fig. 3b). In the case of PVA-containing blends, a substantial modification on surface morphology was found: The ribbons become narrower, less defined and the surface area increased. That was more significant for the blend with low-*M*_*w*_ PVA (Fig. 3c); in fact, for this sample, the surface seemed to suffer erosion, and the ribbonlike structure became much less defined. Both morphologies were different from that of α-cellulose, whose image presented short and disperse fibers (Fig. 4a).

The results of water uptake for all polymers are shown in Fig. 4. The samples that exhibited the highest water absorption capacity were α-cellulose + PVA 27 k and cross-linked α-cellulose; these polymers can sorb 106% and 25%, respectively, more water than the neat α-cellulose. The justification for the latter can be found in the decrease in polymeric structure stability and high adsorption surface. It can be hypothesized that cross-linking will affect both the hydrophilic part, which is positioned along the equatorial plane, and the hydrophobic groups located above and below the axial plane. Therefore, cross-linking will have an impact on the structure of cellulose, affecting its amphiphilicity and, consequently, increasing its ability to interact with water (Yamane et al. 2006; Lindman et al. 2010).

On the other hand, the water uptake showed by cross-linked α-cellulose–PVA polymers is clearly controlled by the PVA molecular weight; i.e., covalent and intermolecular interactions with long-chained PVA led to lower water uptake, as the reduction in porosity (as evidenced, e.g., by comparing Fig. 3c and d) implies a physical barrier against water. In other words, a more diffuse surface morphology, favoring hydrophilicity and porosity at the same time, leads to significantly higher water uptake levels. These results also suggest that PVA cross-linking involved a plasticizer effect, as hypothesized in the discussion above.

### Characterization of BEKP and its derivatives

**Fig. 5:**
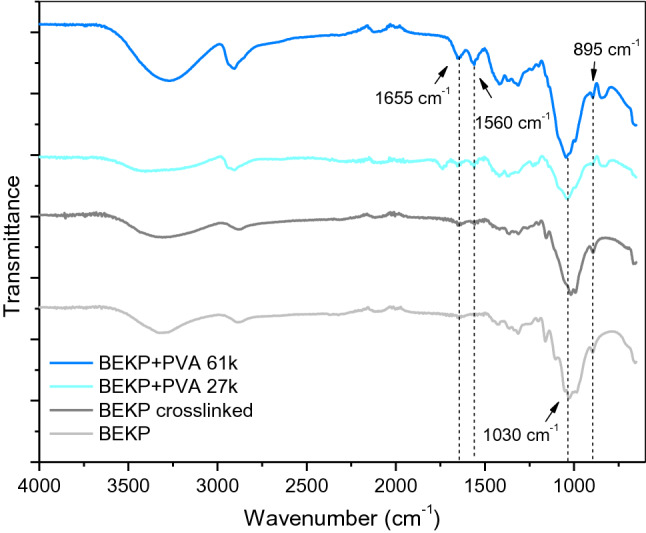
FTIR spectra of BEKP (neat and cross-linked) and BEKP-PVA cross-linked blends

Infrared spectra for BEKP blends were quite similar to those of α-cellulose with slightly shifts in the vibrational mode wavenumbers (Fig. 5). Thus, the absorption bands at 1655 and 1560 cm^−1^ are attributed to the vibrational modes of PVA’s acetate residual groups and MBA (C=O stretching) and N–H rocking from the cross-linker, respectively. Likewise, the prominent band at 1030 cm^−1^ corresponds to C–O–C stretching in cellulose, completely overlapping the C–O stretching of PVA whose absorption band could be located at around 1100 cm^−1^ (hardly distinguishable). As for the absorption at 895 cm^−1^, this is associated with the crystalline structure of cellulose I (Aguado et al. 2019). Solution in NaOH/urea and subsequent regeneration resulted in partial amorphization and/or conversion to cellulose II.

**Fig. 6:**
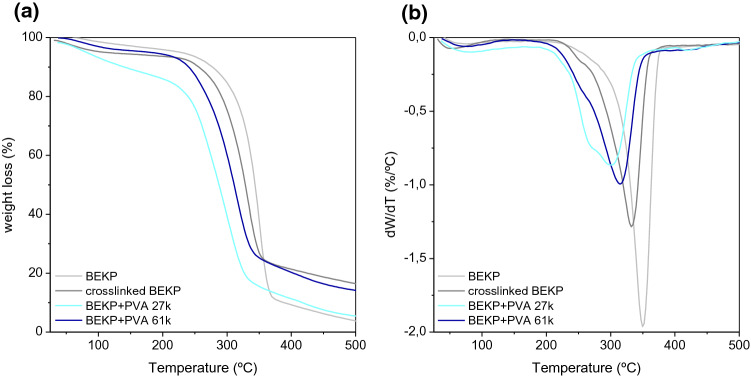
Thermograms (**a**) and DTG curves (**b**) for the neat and cross-linked BEKP materials

The analysis of thermograms from neat and blend BEKP showed slightly, but not negligible, differences between the effect of cross-linker and PVA on the thermal stability of BEKP blends (Fig. 6 and Table 3). The main difference occurred at the *T*_m,2_ transition, where the presence of PVA, in particular that of lower molecular weight, had a significant effect on the thermal stability of the pulp. A decrease of 50 °C on this transition temperature, attributed to cellulose I degradation, decrystallization, or conversion to cellulose II (as discussed in the previous section), may suggest that PVA (and cross-linker at lower extent) acts as an effective plasticizer of BEKP. It is also worth noting that, on contrary to α-cellulose, the degradation steps at lower temperatures (*T*_m,1_) for cross-linked cellulose and BEKP + PVA 27 k can be observed (Tables 2 and 3), indicating the occurrence of non-freezing water to a larger extent.

**Table 3:** Maximum degradation temperatures obtained by thermogravimetric analysis, for BEKP and BEKP + PVA blends, using 0.5 g MBA per gram of polymer

*T* (°C)	*T*_m,1_/°C	*T*_m,2_/°C	*T*_m,3_/°C
BEKP		350	
Cross-linked BEKP	255	332	
BEKP + PVA 27 k	257	300	418
BEKP + PVA 61 k	267	315	407

**Fig. 7:**
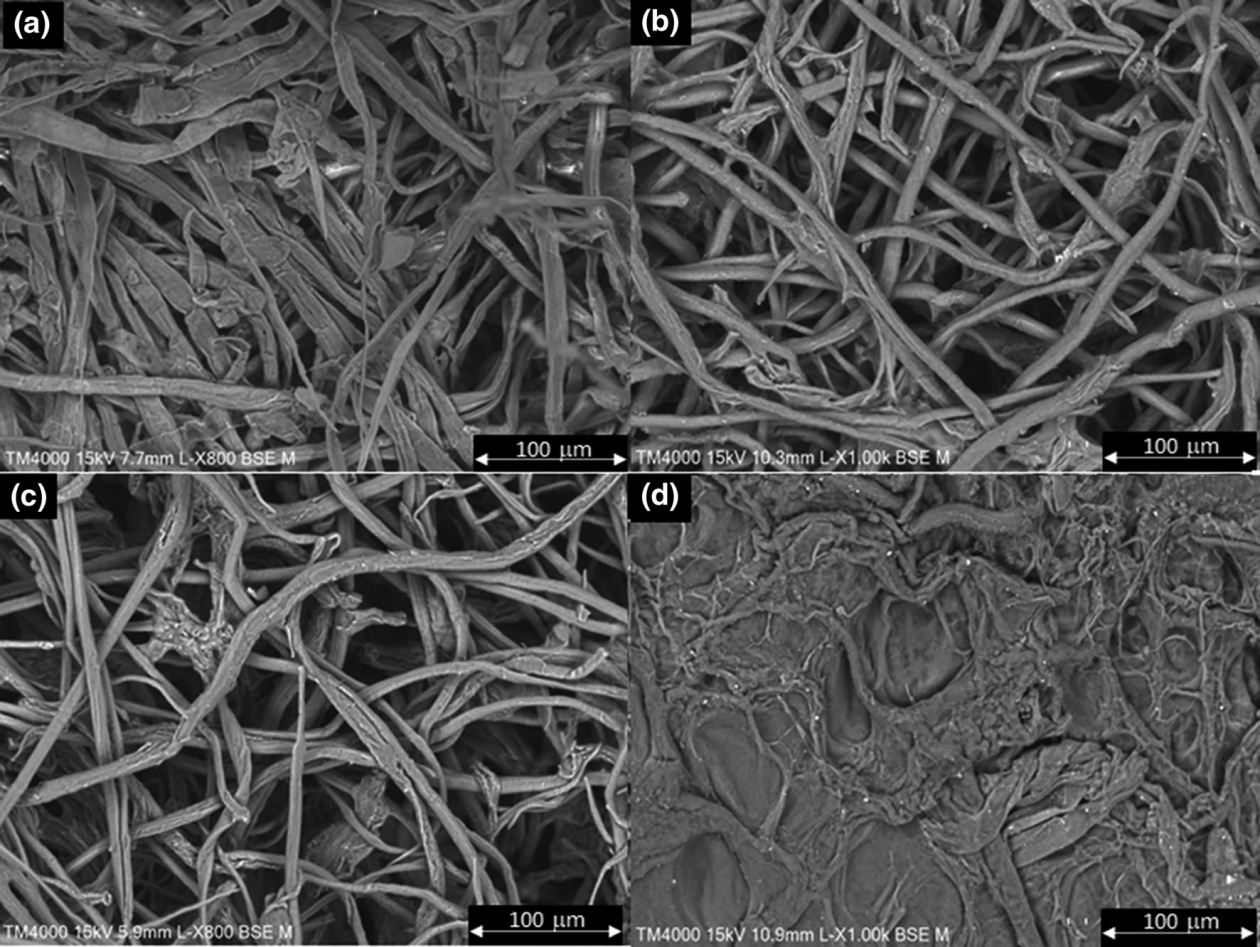
SEM images of BEKP (**a**), cross-linked BEKP (**b**), BEKP + PVA 27 k (**c**) and BEKP + PVA 61 k (**d**). Magnification × 1000

**Fig. 8:**
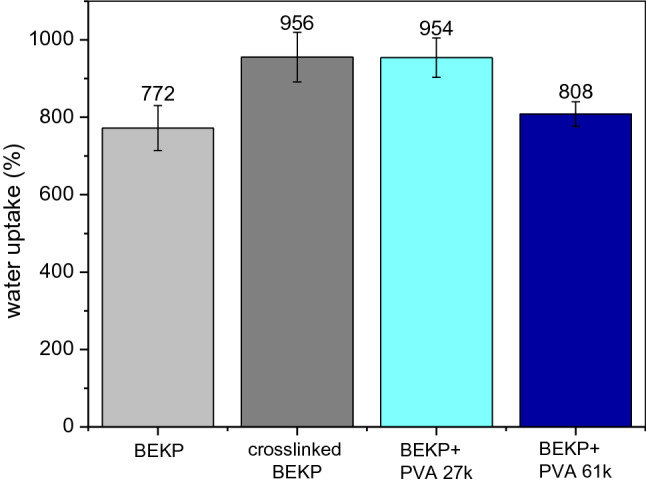
Water uptake for the different BEKP-based polymers

The morphology of the control BEKP (essentially native cellulose, Fig. 7a) consists of fibers with a relatively high aspect ratio, with certain clustering and a high entanglement degree (Chinga-Carrasco et al. 2011). The alkaline treatment and cross-linking changed the surface morphology dramatically, in terms of both fibers by themselves and inter-fiber interactions (Fig. 7b). Regarding the former, the surface and slenderness of fibers resemble that of cellulose I/II hybrids (Yue et al. 2015), due to chain rearrangement in NaOH/urea systems. In parallel, seemingly because intra-fiber cross-linking of cellulose chains overwhelmed inter-fiber cross-linking, a much more porous surface is observed. Similar surface morphology was found for BEKP + PVA 27 k (Fig. 7c). However, for BEKP + PVA 61 k (Fig. 7d), the fiber-like morphology became less clear and the micrograph was dominated by a molten, featureless, and non-porous surface. Unlike PVA of *M*_*w*_ 27,000, that of *M*_*w*_ 61,000 filled the pores instead of coating the fibers. This contributes to the understanding of the low capability of this blend to absorb as much water as the one corresponding to the low-*M*_*w*_ PVA. In fact, the difference in the water uptake of PVA-containing blends was about 18%, or 1.46 g/g in absolute terms. Simultaneously, the water uptake for the cross-linked BEKP and BEKP + PVA 27 k was quite similar. For these reasons, paper strength and other properties were only assessed by using PVA with the lowest molecular weight (Fig. 8).

### Papermaking properties

**Fig. 9:**
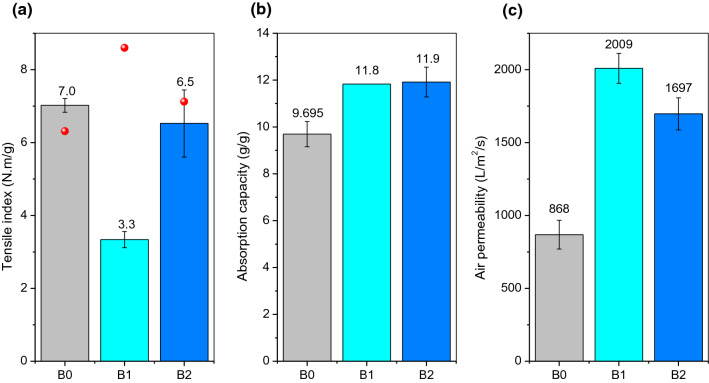
Results of tensile index (**a**), water absorption capacity (**b**), and air permeability (**c**) for the laboratory tissue handsheets of Table 1: B0 (30%BSKP + 70%BEKP); B1 (30%BSKP + 70%MBEKP) and B2 (30%BSKP + 70%MPBEKP). The spheres at panel (a) report the bulk values in cm^3^/g

**Fig. 10:**
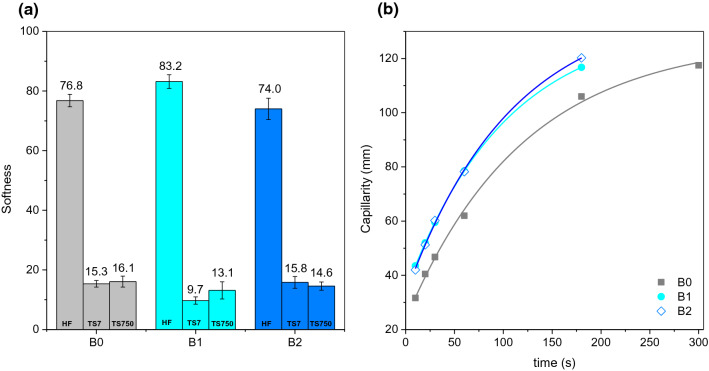
Softness (**a**) and capillarity (**b**) for tissue handsheets, B0 (30%BSKP + 70%BEKP); B1 (30%BSKP + 70%MBEKP) and B2 (30%BSKP + 70%MPBEKP). Solid lines in (**b**) were obtained by fitting Eq. (1) to the experimental data

The results of paper tests performed on laboratory tissue sheets produced with 30% BSKP and 70% modified BEKP (MBEKP) or 70% modified BEKP/PVA 27 k (MPBEKP) are displayed in Figs. 9, 10, and Table 4. Figure 9 shows the properties of different sheets in terms of tensile index, absorption capacity, and air permeability. In all cases, error bars encompass twice the standard deviation.

**Table 4:** Fitting parameters obtained from the capillary kinetics in different paper tissues: B0 (30%BSKP + 70%BEKP); B1 (30%BSKP + 70%MBEKP) and B2 (30%BSKP + 70%MPBEKP), by using Eq. 2

	*H*_0_ (mm)	*H*_e_ (mm)	*t*_c_ (s)	R^2^
B0	24 ± 2	127 ± 4	120 ± 13	0.9970
B1	34.1 ± 0.1	133.9 ± 0.3	102.1 ± 0.6	0.9999
B2	33 ± 1	140 ± 4	111 ± 10	0.9989

The tensile index is one of the most important properties to the paper industry, since the paper web is submitted to tangential stress from its formation to its drying, and tensile resistance is one of the main criteria for quality control (Aguado et al. 2015). It was observed that, in general, the tensile index and the porosity of the surface, as indicated by the transmission rate of air through the sample, followed inverse trends. For B1 (70% cross-linked BEKP), we observed drastic effects on the two properties, in such a way that paper strength decreased upon cross-linking, while water absorption and porosity increased. For the formulations with 70% BEKP + PVA 27 k (B2), the handfeel values and the water absorption capacity were similar and inside the statistic error (random error), even though PVA greatly enhanced water uptake in the case of α-cellulose fibers (Fig. 4).

Cross-linked BEKP, without polymeric additives (B1), resulted in paper sheets with significantly enhanced handfeel (Fig. 10a), but lower tensile index comparatively to the reference sheets. Airflow rate was increased in 131%, i.e., more than doubled (Fig. 9c), which was consistent with the most porous paper having the highest bulk (Fig. 9a). In other words, there was more void volume, which is generally desirable in tissue paper, but the subsequent weaker intermolecular interactions led to worse tensile resistance.

What is remarkable of PVA-containing papers (B2) is that the enhancement of porosity and absorptivity did not compromise paper strength. Overall, taking into account the best balance between all properties shown in Fig. 9, we can conclude that B2 has a consistent improvement in bulk, absorption capacity and air permeability, without detrimental consequences in comparison with the reference sheets. For this safeguarding of strength, the use of fully or almost fully hydrolyzed PVA is recommended. The crippled hydrogen bonding capabilities of PVA with a lower degree of hydrolysis (88%) were detrimental to paper strength and caused loss of non-cross-linked PVA chains to the white water.

The trends in handfeel values were consistent with those of TS7 and TS750 (Fig. 10a). The former parameter, the so-called real softness, is related to the mobility and rigidity of superficial fibers in the *z* direction. Higher values of TS7 mean a rougher surface, due to the obstruction of the analyzer’s blades. Likewise, low values of TS750 indicate “smoothness” (higher influence from topographic variations). Both parameters are used by the TSA algorithm to estimate softness (handfeel) and are thus intimately related to it. They also allow a more detailed analysis over the factors affecting paper softness.

The capillary rise on samples with cross-linked fibers (without and with PVA) showed similar kinetics (Fig. 10b). For a deep evaluation on that, Eq. (2) is fitted to the experimental data. Equation (2) consists on an integrated form of the first-order kinetics equation for capillary rise (Karoglou et al. 2005):

2 $$H = H_{e} - \left( {H_{e} - H_{0} } \right)e^{{ - t/t_{c} }}$$

where *H*_*e*_ and *H*_*0*_ are the equilibrium and correction heights, respectively, and *t*_*c*_ is a constant. Table 4 shows the fitting parameters obtained for all systems. It can be seen that B2 showed the highest *H*_*e*_; however, although *t*_*c*_ values were quite similar for all handsheets, considering the experimental error, B1 showed the slowest rise (Polishchuk and Zaikov 1997; Valente et al. 2005). This can be justified by the porous structure of B1 formulation and by the PVA presence in B2, providing a more hydrophilic interface.

## Conclusions

Cellulose and PVA chains in cotton α-cellulose and in BEKP were successfully cross-linked via MBA, using the latter to prepare laboratory tissue paper handsheets with acceptable tensile resistance. Thermogravimetric analysis, ATR-FTIR spectroscopy, and SEM suggested decrystallization and/or conversion of cellulose I to cellulose II, a plasticizing effect in presence of PVA, modification of the fiber morphology toward a higher specific surface area, and increased porosity of the materials. Water uptake increased by cellulose–MBA–cellulose cross-linking, but with a greater extent when low-*M*_*w*_ (*ca.* 27,000 Da) PVA was cross-linked with cellulose via MBA. Regarding tissue paper laboratory handsheets, while cross-linking alone was enough to attain a water absorption capacity of 11.8 g/g, the addition of PVA was found to be key to avoid a harsh diminishment of paper strength.
